# Changes in T-lymphocyte subsets and risk factors in human immunodeficiency virus-negative patients with active tuberculosis

**DOI:** 10.1007/s15010-020-01451-2

**Published:** 2020-05-29

**Authors:** Kui Li, Renyu Ran, Zicheng Jiang, Chuanqi Fan, Tao Li, Zhiguo Yin

**Affiliations:** 1 Department of Infectious Diseases, Ankang Central Hospital, Ankang, Shaanxi China; 2 Department of Infectious Diseases, Ankang Central Hospital, Hubei University of Medicine, Hubei, China; 3 Department of Pharmacy, Ankang Central Hospital, No. 85, South Jinzhou Road, Hanbin District, Ankang, 725000 Shaanxi China

**Keywords:** Tuberculosis, T-lymphocyte subsets, Risk factors, X-ray computed tomography, Proteins

## Abstract

**Purpose:**

Immune function imbalance is closely associated with the occurrence and development of infectious diseases. We studied the characteristics of changes in T-lymphocyte subsets and their risk factors in HIV-negative patients with active tuberculosis (ATB).

**Methods:**

T-lymphocyte subsets in 275 HIV-negative ATB patients were quantitatively analyzed and compared with an *Mycobacteriumtuberculosis*-free control group. Single-factor and multifactor analyses of clinical and laboratory characteristics of patients were also conducted.

**Results:**

In ATB patients, CD4 and CD8 T-cell counts decreased, and the levels were positively interrelated (*r* = 0.655, *P* < 0.0001). After 4 weeks of antituberculosis treatment, CD4 and CD8 T-cell counts increased significantly but remained lower than in the control group. CD4 and CD8 cell counts were negatively associated with the extent of lesions detected in the chest by computed tomography (all *P* < 0.05). Although not reflected in the CD4/CD8 ratio, CD4 and CD8 cell counts differed between drug-resistant TB patients and drug-susceptible TB patients (*P* = 0.030). The multivariate analysis showed prealbumin, alpha-1 globulin, body mass index, and platelet count were independent risk factors for decreased CD4 cell count (all *P* < 0.05), while age and platelet count were independent risk factors for decreased CD8 cell count (all *P* < 0.05).

**Conclusion:**

CD4 and CD8 T-cell counts showed the evident value in predicting ATB severity. An increase in the CD4/CD8 ratio may be a critical clue of drug resistance in ATB. Although the factors influencing CD4 and CD8 are not identical, our results indicated the importance of serum protein and platelets to ATB patients’ immune function.

**Electronic supplementary material:**

The online version of this article (10.1007/s15010-020-01451-2) contains supplementary material, which is available to authorized users.

## Introduction

Tuberculosis (TB) is a major infectious disease that seriously threatens human health. One-third of the world’s population is infected with *Mycobacterium tuberculosis* (*Mtb*). Among infectious diseases, TB produces the highest single-cause mortality and is a worldwide public health issue that cannot be ignored. The pathogenesis of TB is complex. Compromised immunity is a major risk factor for TB infection [1], while chronic inflammation leads to T-cell dysfunction [2]. The synergistic effect of these factors increases the risk of TB infection and accelerates disease progression.

Attempts to develop alternative novel methods of adjuvant therapy for TB infections have focused on determining the characteristics of the changes in immune function that occur during TB infection and their risk factors. Consensus has emerged that *Mtb* downregulates the activation of CD4 T-cells through secreted-protein-mediated interference with T-cell proximal and downstream signals, leading to Th1/Th2 imbalance [3] and eventually inducing low expression of CD4 [4, 5]. However, reports on the changes in CD8 T-cells have been inconsistent. Virginie et al. [6, 7] reported an increase in CD8 T-cell numbers, whereas Athman et al. [8, 9] showed a decrease. In terms of risk factors, previous studies have shown that factors including human immunodeficiency virus (HIV), diabetes [10, 11], parasitic infections [12, 13], cavities [14, 15], low 1.25 (OH) 2D3 [16, 17], and high *Mtb* load [18] can lead to deficiencies in the number and function of T lymphocytes. In addition to these inconsistent results, most existing studies are small-sample, single-factor analyses. Thus, there is an urgent need for in-depth studies in this field.

In this study, we compared the changes in the numbers of CD4 and CD8 T-cells in patients with HIV-negative active tuberculosis (ATB), examined the factors associated with these changes by regression analysis using the optimal scaling method, and further evaluated the degree of correlation. The results provide a foundation for more accurately understanding the changes in immune function in TB patients and providing early intervention in high-risk cases in future clinical practice.

## Methods

### Study design

We retrospectively analyzed patients with pathogenically positive TB who were continuously hospitalized in Ankang Central Hospital. During the same period, individuals without *Mtb* infection and clinical symptoms were included as the control. The data were collected between October 1, 2016, and June 30, 2018. A total of 26 variables based on five aspects (the patients’ basic condition, routine examination, protein electrophoresis, bacteriology, and imaging) were collected and analyzed.

### Inclusion and exclusion criteria

Inclusion criteria [19]: (1) positive *Mtb*-deoxyribonucleic acid/ribonucleic acid test accompanied by suspected TB symptoms; (2) one sputum specimen smear-positive for acid-fast staining or culture-positive for *Mtb*, in addition to chest computed tomography scans that detected lesions consistent with ATB (which might or might not be accompanied by suspected TB symptoms); (3) the patient’s T-lymphocyte subsets had been examined, and the results were available.

Exclusion criteria: (1) individuals who had bilirubin levels ≥ 2 times the upper limit of normal; (2) individuals who were positive for HIV antibody; (3) individuals with nontuberculous mycobacterial lung disease; (4) individuals with concurrent cirrhosis or tumors; and (5) individuals who were taking immunosuppressants.

### T-lymphocyte subset assays

T-lymphocyte subset assays were performed using a flow cytometer detection platform (Mindray flow cytometer BriCyte E6, Shenzhen Mindray Bio-Medical Electronics Co., Ltd. Shenzhen, China) and a lymphocyte detection kit (BD Biosciences, Franklin Lakes, NJ, USA). The samples were stored and transported at room temperature (20–25 °C). The samples were assayed within 48 h after collection, and the flow cytometric examination was conducted within 24 h after staining. The main steps were as follows. (1) Fasting venous blood (5 mL) was collected and mixed thoroughly with the anticoagulant EDTA (ethylenediaminetetraacetic acid). (2) The CD3/CD8/CD45/CD4 multitest reagents were added to test tubes (20 μL per tube). (3) Fifty microliters of EDTA-anticoagulated venous blood was added to the bottom of each test tube (the blood sample was not permitted to touch the wall of the test tube). (4) The mixture was thoroughly mixed on a vortexer and incubated for 15 min at room temperature in the dark. (5) After the addition of 450 μL of hemolysin to each test tube, the mixture was mixed gently on a vortex mixer and incubated for 15 min at room temperature in the dark. (6) Automated detection was performed on a flow cytometer.

### Measurement of *Mtb* load and other variables

*Mtb* load was determined in accordance with the “Diagnostic criteria and principles of management of infectious pulmonary tuberculosis” [19] (Supplementary Table 1) using Roche solid culture medium. The patients were not subjected to repeated analysis. Body mass index (BMI) was calculated as the patient’s body weight (kg) divided by the square of his or her height (m). The patients’ weights, heights, medical histories, and personal histories were collected by the medical staff. The diagnostic criterion for diabetes was random blood glucose ≥ 11.1 mmol/L or fasting blood glucose ≥ 7.0 mmol/L [20]. The extent of the disease was assessed by chest computed tomography (EDCT) in accordance with the standards of the National Tuberculosis Association of the United States [21] (Supplementary Table 2). Whole blood cell counts were performed using a Sysmex XN-9000 automatic blood fluid analyzer and the appropriate reagents. Serum protein electrophoresis was performed on the Sebia Capillarys 2 Flex Piercing platform with the appropriate reagents (Pare Technologique Leonardo da Vinci, CP8010-Lisses 91,008, Evry, CEDEX, France).

### Statistical analysis

The normality of the data was assessed using the moment method. The distributions of the data are described by the median and interquartile range (IQR). In univariate analysis, between-group comparisons of dichotomous variables were performed using the Wilcoxon rank-sum test, and between-group comparisons of grades and measurement data were conducted using Spearman correlation analysis. Collinearity was defined as a correlation coefficient greater than 0.7 [22] or a tolerance value lower than 0.1 between two variables before data transformation. The variables that caused collinearity were culled. Missing values were replaced with the series mean. The statistically significant variables in the univariate analysis were subjected to optimal scale regression analysis. Finally, correlation analyses were performed using linear regression equations. All analyses were performed using SPSS 22.0 (IBM Corp., Armonk, NY, USA) and GraphPad Prism 8.0.2 (GraphPad Software, La Jolla, CA, USA) software. *P* values of less than 0.05 indicated statistically significant differences.

## Results

### Participant characteristics

**Fig. 1:**
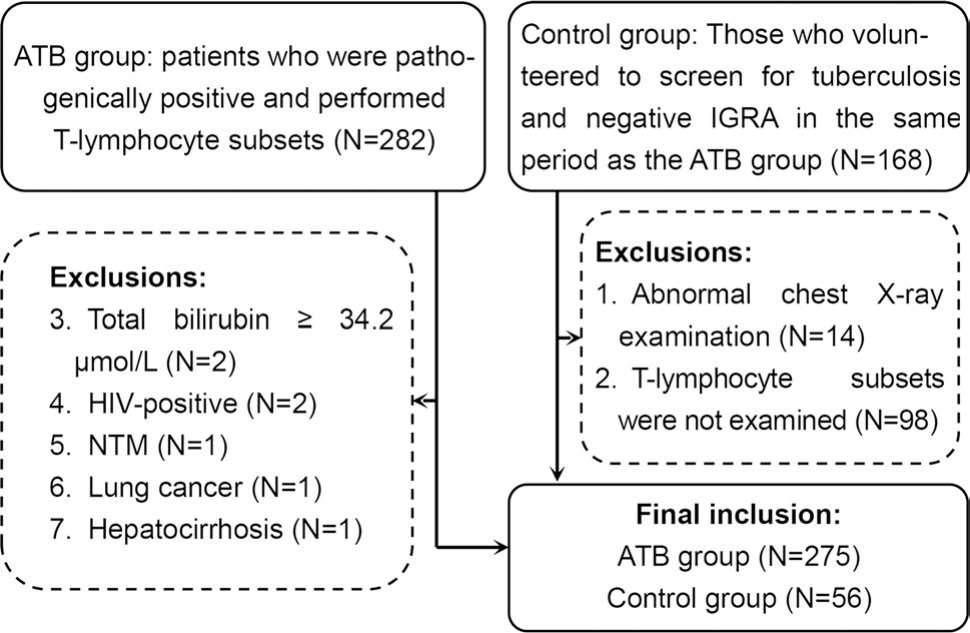
Flowchart of the study population. *ATB* active tuberculosis, *HIV* human immunodeficiency virus, *IGRA* interferon gamma release assay, *NTM* nontuberculous mycobacteria

A total of 450 individuals were screened for study eligibility, and a total of 331 (73.5%) were included in the study, including 275 confirmed cases of ATB (Fig. 1). The characteristics of the participants are shown in Table 1. In the ATB group, 89.5% (246/275) received the standard nationally recommended chemotherapy regimen. After 4 weeks, 66.2% (182/275) of the patients completed a second test for T-lymphocyte subsets. There was no significant difference in gender or age between the control group and the ATB group (*χ*^2^ = 2.280, *P* = 0.131 and *Z* = 1761.50, *P* = 0.689, respectively).

**Table 1:** Demographic and laboratory characteristics of the study participants

Characteristics	Control group (*N* = 56)	ATB group (*N* = 275)
Male sex, No. (%)	39 (69.6)	217 (78.9)
Age, years, M (IQR)	51 (38–64)	54 (38–66)
At admission		
CD4 T-cell count, cells/μL, M (IQR)	855.5 (689.8–982.2)	300.0 (203.0–437.0)
CD8 T-cell count, cells/μL, M (IQR)	560.0 (465.8–730.7)	250.0 (164.0–378.0)
CD4/CD8 ratio, M (IQR)	1.40 (0.99–1.75)	1.19 (0.89–1.70)
Anti-TB regimen, No. (%)		
H + R + Z + E	NA	144 (52.4)
H + R + Z + E + Lfx/Am	NA	76 (27.6)
H + R + Lfx + E^a^	NA	19 (6.9)
R + Z + E + Lfx + Am	NA	14 (5.1)
H + Z + E + Lfx + Am	NA	12 (4.4)
Other^b^	NA	10 (3.6)
At the end of the 4th week after anti-TB^c^		
CD4 T-cell count, cells/μL, M (IQR)	NA	517.0 (361.3–654.5)
CD8 T-cell count, cells/μL, M (IQR)	NA	367.5 (237.5–555.8)
CD4/CD8 ratio, M (IQR)	NA	1.35 (0.93–1.76)

*Am* amikacin, *ATB* active tuberculosis, *E* ethambutol, *H* isoniazid, *IQR* interquartile range, *Lfx* levofloxacin, *M* median, *NA* not applicable, *R* rifampicin, *TB* tuberculosis, *Z* pyrazinamide

^a^Regimen used when drug-sensitive patients could not tolerate the side effects of pyrazinamide

^b^Individualized treatment for multidrug- or extensively drug-resistant tuberculosis

^c^Data from 182 patients who completed re-examination of T-lymphocyte subsets

### Changes in CD4 and CD8 T-cell numbers

**Fig. 2:**
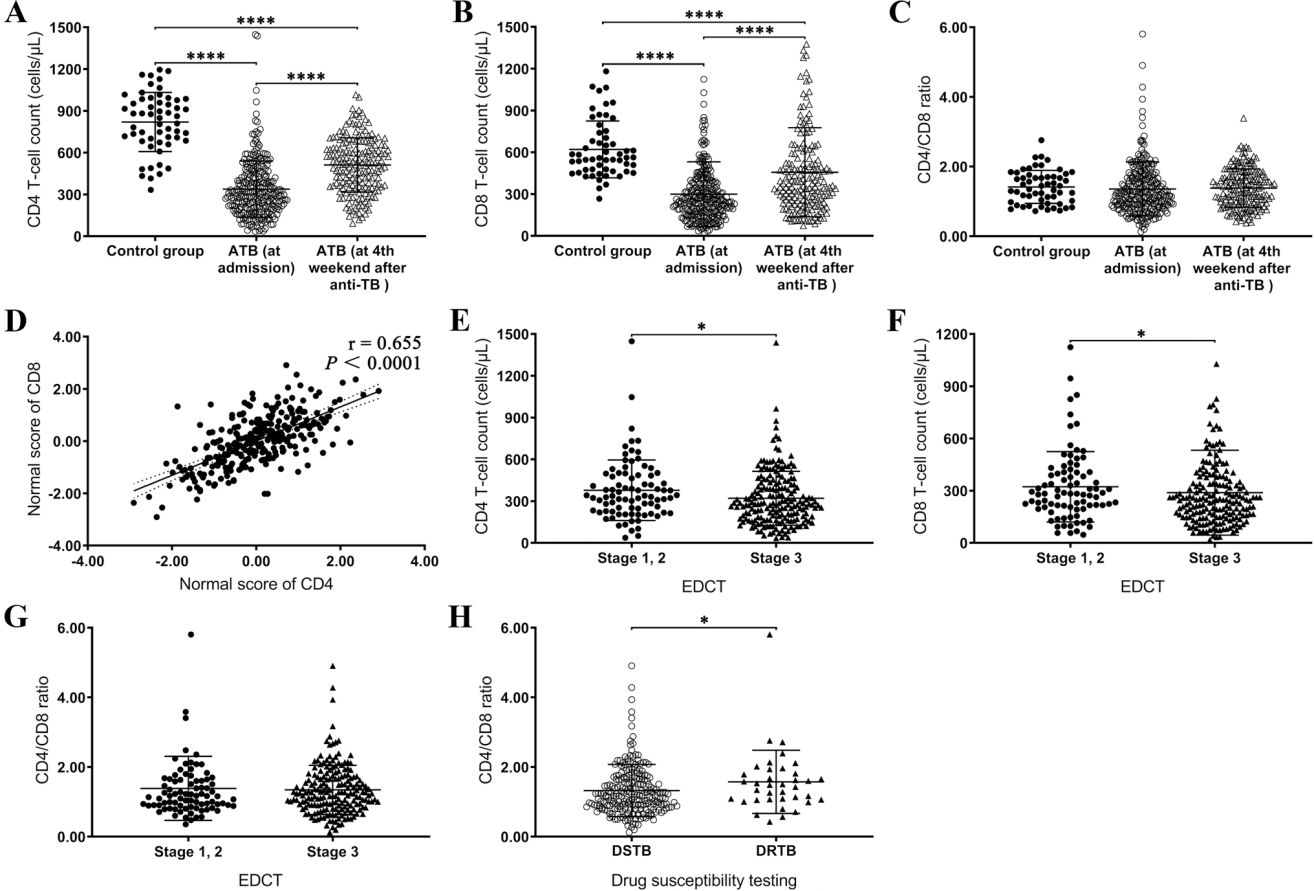
Comparison of CD4 and CD8 T-cell counts at the time of admission with those of patients in the control group and those of patients after anti-TB treatment (**a-c**). Correlation analysis of CD4 and CD8 T-cell counts in the ATB group (**d**). The normal distribution values of CD4 and CD8 T-cell counts were converted using Rankit’s formula; CD4 and CD8 T-cell counts were associated with the extent of the disease as assessed by chest computed tomography (**e–g**). Comparison of CD4/CD8 ratios in patients with drug-susceptible and drug-resistant TB (**h**). *P < 0.05, ****P < 0.0001. *ATB* active tuberculosis, *BMI* body mass index, *EDCT* extent of the disease as assessed by chest computed tomography, *DRTB* drug-resistant tuberculosis, *DSTB* drug-susceptible tuberculosis, *TB* tuberculosis

Compared with the control group, the numbers of CD4 T-cells and CD8 T-cells in the ATB group were lower (*Z* = 850.50, *P* < 0.0001 and *Z* = 1504.50, *P* < 0.0001, respectively; Fig. 2a, b). After 4 weeks of anti-TB treatment, the numbers of CD4 and CD8 T-cells increased markedly in the ATB group compared with the cell numbers before treatment (*Z* = 37,712.50, *P* < 0.0001 and *Z* = 33,919.00, *P* < 0.0001, respectively; Fig. 2a, b) but were still below the corresponding levels in the control group (*Z* = 1498.50, *P* < 0.0001 and *Z* = 2605.00, *P* < 0.0001, respectively; Fig. 2a, b). There were no significant differences in the CD4/CD8 ratio among the groups (all *P* > 0.05; Fig. 2c). In the HIV-negative ATB group, CD4 and CD8 cell numbers were positively correlated with each other (*r* = 0.655, *P* < 0.0001; Fig. 2d).

### CD4 and CD8 are associated with EDCT

Decreases in CD4 and CD8 T-cell counts at the time of admission were negatively correlated with the EDCT results (*r* = − 0.147, *P* = 0.015 and *r* = − 0.127, *P* = 0.035, respectively; Table 2 and Fig. 2e, f), but the CD4/CD8 ratio showed no correlation with EDCT (*r* = 0.028, *P* = 0.647) (Table 2 and Fig. 2g).

**Table 2:** Univariate analysis of T-lymphocytes (nominal and ordinal variables)

Factor	*N*	CD4 T-cell count		CD8 T-cell count		CD4/CD8 ratio	
		Cells/μL, median (IQR)	*P* value	Cells/μL, median (IQR)	*P* value	Median (IQR)	*P* value
Sex							
Female	58	322.0 (202.3–464.8)	0.501	253.5 (143.8–387.8)	0.956	1.24 (0.93–1.67)	0.511
Male	217	288.0 (200.5–436.0)		249.0 (164.0–377.5)		1.17 (0.87–1.68)	
Smoking^a^							
No	104	317.0 (210.0–462.8)	0.208	262.0 (176.3–384.3)	0.168	1.23 (0.81–1.65)	0.666
Yes	171	285.0 (190.0–427.0)		240.0 (148.0–355.0)		1.18 (0.90–1.71)	
Dust exposure^a^							
No	194	297.5 (189.0–438.0)	0.481	249.5 (154.0–389.3)	0.784	1.17 (0.85–1.66)	0.312
Yes	81	302.0 (218.5–441.0)		259.0 (165.5–344.5)		1.27 (0.91–1.73)	
Previously treated cases							
No^b^	194	307.5 (213.0–433.5)	0.062	262.0 (174.0–383.5)	0.087	1.21 (0.90–1.66)	0.854
Yes^c^	81	271.0 (153.5–455.0)		224.0 (133.5–348.0)		1.17 (0.84–1.80)	
Cavitation							
No	95	313.0 (174.0–437.0)	0.818	240.0 (129.0–382.0)	0.266	1.23 (0.95–1.65)	0.386
Yes	180	290.5 (205.3–442.5)		252.0 (175.3–377.8)		1.17 (0.84–1.68)	
Extrapulmonary tuberculosis							
No	227	311.0 (203.0–459.0)	0.069	259.0 (166.0–382.0)	0.140	1.19 (0.87–1.70)	0.897
Yes	48	267.0 (185.8–378.8)		231.5 (141.8–323.8)		1.21 (0.93–1.55)	
Diabetes mellitus							
No	247	309.0 (197.0–445.0)	0.541	250.0 (159.0–377.0)	0.664	1.22 (0.90–1.66)	0.462
Yes	28	263.5 (207.8–414.8)		255.0 (185.3–402.0)		0.97 (0.76–1.73)	
Drug resistant tuberculosis							
DSTB	238	297.5 (193.5–439.0)	0.398	256.5 (157.8–382.0)	0.442	1.17 (0.85–1.66)	**0.030**
DRTB	37	316.0 (248.5–447.5)		221.0 (165.5–358.0)		1.49 (1.07–1.87)	
Grade^d^							
DNA/RNA positive	57	323.0 (213.0–508.0)	**0.043**	263.0 (169.0–385.5)	0.152	1.18 (0.93–1.67)	0.771
+	15	393.0 (168.0–653.0)		329.0 (213.0–431.0)		1.17 (0.87–1.60)	
1 +	67	274.0 (186.0–466.0)		262.0 (148.0–379.0)		1.09 (0.78–1.75)	
2 +	51	331.0 (217.0–437.0)		240.0 (150.0–343.0)		1.27 (0.90–1.84)	
3 +	55	278.0 (158.0–369.0)		227.0 (147.0–272.0)		1.29 (0.99–1.79)	
4 +	30	279.0 (215.5–394.5)		262.0 (175.5–446.5)		0.98 (0.74–1.51)	
EDCT							
Stage 1 (minimal/mild)	1	538.0	**0.015**	945.0	**0.035**	0.57	0.647
Stage 2 (moderate)	83	331.0 (225.0–483.0)		283.0 (205.0–405.0)		1.17 (0.89–1.64)	
Stage 3 (advanced)	191	280.0 (174.0–425.0)		245.0 (148.0–343.0)		1.24 (0.89–1.71)	

Statistically significant associations are marked in bold

*DNA* deoxyribonucleic acid, *DRTB* drug-resistant tuberculosis, *DSTB* drug-susceptible tuberculosis, *EDCT* extent of the disease as assessed by chest computed tomography, *IQR* interquartile range, *RNA* ribonucleic acid

^a^Smoking or dust exposure for at least 3 months before a diagnosis of pulmonary tuberculosis

^b^New cases are defined as patients who had not started anti-TB treatment or had been on anti-TB treatment for < 1 month

^c^Previously treated cases are defined as patients who received anti-TB treatment ≥ 1 month in the past

^d^*Mycobacterium tuberculosis* load grading before treatment

### Correlation between the CD4/CD8 ratio and drug-resistant TB

Compared with patients with drug-susceptible TB, the number of CD8 T-cells showed a greater decrease in patients with drug-resistant TB, but the difference was not statistically significant (*Z* = 4057.00, *P* = 0.442; Table 2). However, it is worth noting that the increase in the CD4/CD8 ratio in the drug-susceptible TB patients was statistically significant (*Z* = 5378.00, *P* = 0.030) (Table 2 and Fig. 2h).

### Independent risk factors for CD4 and corresponding correlation coefficients

**Fig. 3:**
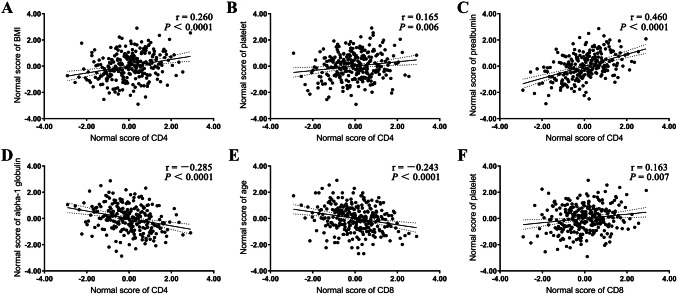
Correlation analysis of independent risk factors for decreases in CD4 and CD8 T-cell counts. The normal distribution values of CD4 and CD8 T-cell counts were converted using Rankit’s formula. *BMI* body mass index

The albumin/globulin ratio was excluded from the multivariate analysis due to collinearity, as its correlation coefficient with albumin was greater than 0.7 (|*r*| = 0.761). There were no variables with tolerance values less than 0.1 (Supplementary Table 3). The optimal scale regression analysis showed that BMI, platelet count, prealbumin (PA) level, and alpha-1 globulin level were independent risk factors for decreased CD4 T-cell counts (all *P* < 0.05; Table 3). The correlation analysis showed that CD4 T-cell count was positively correlated with BMI, platelet count, and prealbumin level (*r* = 0.260, *P* < 0.0001; *r* = 0.165, *P* < 0.006 and *r* = 0.460, *P* < 0.0001, respectively; Fig. 3a–c) and negatively correlated with alpha-1 globulin level (*r* = − 0.285, *P* < 0.0001; Fig. 3d).

**Table 3:** Univariate and multivariate analysis of T-lymphocytes

Factor	Missing data		Median (interquartile rang)^a^	CD4 T-cell count		CD8 T-cell count		CD4/CD8 ratio	
	*N*	(%)		*P* value^b^	*P* value^c^	*P* value^b^	*P* value^c^	*P* value^b^	*P* value^c^
Drug-resistant tuberculosis	0	(00.0)	NA	0.398		0.442		**0.030**	0.061
Grade	0	(00.0)	NA	**0.043**	0.061	0.152		0.771	
EDCT	0	(00.0)	NA	**0.015**	0.628	**0.035**	0.134	0.647	
Age, years	0	(00.0)	54.0 (38.0–66.0)	**< 0.001**	0.214	**< 0.001**	**0.001**	0.484	
Body mass index, kg/m^2^	8	(2.9)	19.1 (17.5–21.1)	**< 0.001**	**0.035**	**0.001**	0.254	0.345	
Duration of symptoms, days	2	(0.7)	90.0 (30.0–730.0)	**0.007**	0.149	0.260		0.102	
White blood cell, × 10^9^/L	0	(00.0)	6.8 (5.6–8.7)	**0.049**	0.068	0.155		0.155	
Hemoglobin, g/dL	0	(00.0)	11.7 (10.4–13.3)	**< 0.001**	0.156	**0.003**	0.220	0.382	
Platelet, × 10^9^/L	0	(00.0)	260.0 (193.0–337.0)	**0.026**	**0.005**	**0.027**	**0.011**	0.941	
Erythrocyte sedimentation rate, mm/H	26	(9.5)	52.0 (29.0–75.5)	**0.002**	0.958	0.220		**0.006**	0.294
Prealbumin, mg/L	35	(12.7)	132.5 (69.3–196.8)	**< 0.001**	**0.020**	**< 0.001**	0.801	**< 0.001**	**0.035**
Albumin, g/L	0	(00.0)	31.8 (26.6–35.7)	**< 0.001**	0.069	**< 0.001**	0.093	**0.030**	0.108
Globulin, g/L	3	(1.1)	32.6 (28.8–36.4)	0.940		0.759		0.426	
Albumin/globulin ratio^d^	3	(1.1)	0.9 (0.8–1.2)	**< 0.001**		**0.006**		**0.033**	
Alpha-1 globulin, g/L	33	(12.0)	4.7 (3.6–6.0)	**< 0.001**	**0.003**	0.085		**0.001**	**0.033**
Alpha-2 globulin, g/L	33	(12.0)	7.7 (6.4–8.7)	0.677		0.429		0.184	
Beta-1 globulin, g/L	33	(12.0)	3.7 (3.2–4.2)	**< 0.001**	0.689	**0.008**	0.545	**0.025**	**0.035**
Beta-2 globulin, g/L	33	(12.0)	3.6 (3.1–4.4)	0.211		0.336		0.804	
Gamma globulin, g/L	33	(12.0)	12.6 (10.1–15.3)	0.378		0.517		0.789	

Statistically significant associations are marked in bold

*EDCT* extent of the disease as assessed by chest computed tomography, *NA* not applicable

^a^Data are presented as the value of the factor

^b^Data are presented as the results of univariate Spearman’s rho correlation coefficient analysis

^c^Data are presented as the results of multivariate optimal scale regression analysis

^d^The correlation coefficient with albumin was greater than 0.7; therefore, albumin/globulin ratio was excluded from the multivariate analysis

### Independent risk factors for CD8 and corresponding correlation coefficients

Age and platelet count were independent risk factors for CD8 T-cell count (all *P* < 0.05; Table 3). CD8 T-cell count was negatively correlated with age (*r* = − 0.243, *P* < 0.0001; Fig. 3e) but positively associated with platelet count (*r* = 0.163, *P* = 0.007; Fig. 3f).

## Discussion

In this study, through comparing and analyzing T lymphocytes in HIV-negative ATB patients, we characterized changes in CD4 and CD8 T-cell numbers and their relationships with EDCT. We also identified the major factors influencing the numbers of CD4 and CD8 T-cells and the strengths of their correlations.

The results of our study suggest that both CD4 and CD8 T-cell counts are lower in patients with ATB. CD4 T helper cells promote the proliferation and differentiation of immune cells such as CD8 T-cells [1] and B-cells and amplify the immune response, resulting in inhibition of the growth of *Mtb*. CD4 T-cells play a leading role in the immunity of ATB patients, as shown by many convincing studies worldwide [23] and confirmed by our study, as CD4 and *Mtb* bacterial load were negatively correlated (*r* = − 0.122, *P* = 0.043; Table 2). CD8 T-cells can kill *Mtb* by particle-mediated effects (such as perforin, granzyme, and others) and can induce cell apoptosis through interaction between Fas and Fas ligands. Our study showed that the number of CD8 T-cells decreased in HIV-negative ATB patients and was positively correlated with CD4 T-cell number, supporting the reduction in CD8 T-cells in HIV-negative ATB and indicating that the reduction in the number of CD4 and CD8 T-cells can be partially reversed by anti-TB treatment. The CD4/CD8 ratio did not reflect the characteristics of the changes in the immune functions of HIV-negative patients with ATB; there was no detectable difference in the CD4/CD8 ratios in the control group and in the ATB group before or after treatment. Furthermore, according to multifactor analysis, the independent factors influencing the CD4/CD8 ratio overlapped with the risk factors for CD4 (Table 3). However, in patients with drug-resistant TB, as the number of CD8 T-cells decreased further, the corresponding increase in the CD4/CD8 ratio was statistically significant, suggesting that the observed depletion of CD8 T-cells may be related to the development of drug resistance. It is speculated that in addition to the drug resistance mechanism of *Mtb* itself, this phenomenon is mainly related to an insufficient number of CD8 T-cells, which leads to weakened secretion of granzyme and perforin, decreased cytolytic activity and the inability to clear intracellular pathogens [24]. Further study is needed to determine which occurs first, the decline in T-lymphocyte counts or the decline in activity after *Mtb* infection.

The results of this study demonstrated that lower CD4 and CD8 T-cell counts were associated with greater extent of lesions in the patients, suggesting that immune function declines with increasing severity of the disease. This finding differs from that of an earlier report [25], possibly due to differences in sample sizes, geographical environments, and ethnic backgrounds studied. No significant changes in CD4/CD8 ratios were detected, and in peripheral blood, CD4 and CD8 are more sensitive markers than the CD4/CD8 ratio. Changes in CD4 and CD8 T-cells were not associated with any concurrent extrapulmonary TB, and the influences of the changes in previously treated cases and in new cases were marginal. In the in-depth analysis, we found that the incidence of severe lesions was higher in previously treated cases (*χ*^2^ = 13.392, *P* < 0.0001; Supplementary Table 4). Thus, it is clear that CD4 and CD8 T-cell counts have certain value for predicting the severity of TB lesions in EDCT. However, severe lesions are also associated with a greater likelihood of treatment failure.

T-cell proliferation and gamma interferon synthesis decrease with age [26]. These changes are detrimental to the body's defenses and decrease the protective response against TB. Patients age 61 and over accounted for 36.73% (101/275) of the patients in the present study. The univariate analysis found that age was significantly negatively correlated with CD4 and CD8 T-cell counts, while the multivariate analysis showed that age was the main factor that negatively influenced CD8 T-cell count. The above findings indicate that a decrease in CD8 cytotoxic T-cell-mediated protective immunity against *Mtb* might cause an increase in the incidence of TB and reactivation of previous infection. The findings are consistent with the results of Chen et al. [27], who showed that CD8 T-cell depletion may result in *Mtb* reinfection.

Accumulating evidence suggests that platelets are the key effector cells in host regulation of inflammatory responses and that they contribute to the initiation and transmission of local and systemic inflammatory processes [28]. Platelets stimulate T-lymphocyte adhesion by secreting the key chemical inducer RANTES (regulated via activation, normal T-cell expressed and secreted, also known as CCL5), thereby regulating T-lymphocyte function [29]. The present study found that changes in the number of platelets were positively correlated with CD4 and CD8 T-cell counts. We were unable to retrieve any clinical data related to this finding from previous studies on ATB.

BMI, hemoglobin, PA, and albumin are indicators that reflect the nutritional status of patients. The negative effects of malnutrition on cellular immunity have been firmly established [30]. The underlying mechanisms may involve impaired antigen-presenting cell function [31] and inhibition of glucose metabolism-dependent T-cell activation [32, 33], both of which reduce the production of protective cytokines [34]. In tuberculosis patients, anemia is related to the overexpression of pro-inflammatory factors that inhibit erythropoietin and to changes in iron metabolism [35]. Due to the correlations of BMI and hemoglobin with CD4, BMI may be a potential indicator reflecting CD4 counts under limited resources [36–38]. These quantitative indicators demonstrate a positive correlation between malnutrition and immune status in TB, and it is possible that malnutrition increases the risk of immune impairment more robustly than does diabetes. Among the malnutrition indicators studied, the PA level was the most important. These findings suggest that the use of existing antituberculosis drugs and improving the nutritional status of the general population may be key TB control strategies.

The alpha-1 globulins mainly consist of alpha-1 antitrypsin (AAT), alpha-1-acid glycoprotein (AGP), alpha-1 lipoprotein, and alpha-fetoprotein. AAT and AGP are acute-phase response proteins [39, 40]. In ATB, alpha-1 globulin performs the functions of both AGP and AAT. In a study conducted by Zhang et al. the most significant differential protein peak in serum was identified as AGP [41], and the most important cell sources included alveolar macrophages and type II alveolar cells [42]. In the present study, the alpha-1 globulin level exceeded the upper limit of normal (4.36 g/L) in 58.26% (141/242) of the study sample, consistent with the results of recent studies [43, 44]. The multivariate analysis showed that alpha-1 globulin level was negatively correlated with quantitative changes in CD4 T-cell count. In contrast, the erythrocyte sedimentation rate, which is also a marker of the inflammatory response, was eliminated from the analysis due to its nonsignificant effect. Moreover, the alpha-1 globulin level was more important than both BMI and platelet count. These clinical data suggest that alpha-1 globulin can inhibit cell-mediated immune responses (Fig. 3d) and promote *Mtb* growth and disease progression [42]. Thus, the alpha-1 globulin level deserves further investigation as a potential marker.

Our research demonstrates that *Mtb* load is negatively correlated with the number of CD4 cells, and after treatment, T lymphocytes may increase, possibly because the expression of the *Mtb* immunodominant antigen depends on the level of virulence protein secreted by *Mtb* entering the cytoplasm [45]. These results are consistent with those of previous studies showing that high antigen levels consistently lead to T-cell failure [18, 46]. However, we found that among patients with high *Mtb* load (*Mtb* load ≥ 3 +), 18.82% (16/85) of patients still had CD4 ≥ 404 cells/μL, and 56.47% (48/85) of patients had CD8 ≥ 220 cells/μL. These patients did not show decreases in the number of lymphocytes, which may be related to the upregulation of the expression of T-cell inhibitory receptors (such as programmed death-1, T-cell immunoglobulin and mucin domain-containing protein-3, and lymphocyte-activated gene-3). These T-cell inhibitory receptors not only negatively regulate the proliferation of reactive cells but also affect their function and cause immune paralysis [47].

In patients with a history of previous treatment, the T-lymphocyte count was even lower. Although the statistical results were marginal, the duration of symptoms before admission was negatively correlated with the CD4 count, suggesting that the continued presence of bacterial antigens leads to lymphocyte failure. This finding is consistent with the importance of the timing of the immune response in human viral infectious diseases [48].

The main component of white blood cells (WBC) is neutrophils. Neutrophils can carry antigens from peripheral sites into lymph nodes and bone marrow and promote the generation of Th1, Th17, and CD8 memory responses [49–51]. The release of neutrophil extracellular traps (NETs) can reduce the activation threshold of T-cells [52] and promote the activation of naïve antigen-specific CD4 T-cells [53]. In addition, the production of granulocyte–macrophage colony-stimulating factors by T-cells during *Mtb* infection can enhance the host’s resistance to *Mtb* [54]. This study found a positive correlation of WBC with CD4 but no statistically significant correlation with CD8, suggesting that WBC is one of the activation factors affecting CD4 cells.

The main proteins in the beta-1 globulin fraction are transferrin, hemopexin, beta-lipoprotein, and complement C4. Transferrin and hemopexin are involved in the transport and storage of iron, and they often decline together with albumin during acute-phase reactions and malnutrition. Previously, Minchella et al. [55] found that low transferrin levels are a risk factor for the development of active tuberculosis in people with latent tuberculosis infection. In this study, all β1 globulin proteins had various effects on T lymphocytes, suggesting that β1 globulin may promote tuberculosis activities by acting on the body’s immune function via the following routes: transferrin and hemopexin may decrease, thereby reducing transferrin-Fe^3+^ entering the bone marrow; during *Mtb* infection, pathogens may obtain iron from host iron carrier proteins via the synthesis of high-affinity iron chelators and expression of the glyceraldehyde-3-phosphate dehydrogenase receptor on the cell surface [56]; the patient’s iron deficiency leads to inhibition of lymphocyte DNA synthesis, abnormal proliferation, and differentiation of T lymphocytes, and a reduced antibacterial effect of macrophages and granulocytes [57, 58]. β-Lipoprotein can decrease with the loss of plasma protein, and its final change depends on compensatory synthesis by hepatocytes, which is unpredictable. Complement C4 increases in inflammation and infection, and the increased complement C4 in this study may offset and mask the correlation between transferrin and T-lymphocyte count. The relationship between lymphocytes and transferrin warrants further study. Regulating the imbalance of iron in the body and blocking the ion supply of *Mtb* may be a potential strategy to rescue drug-resistant tuberculosis and improve the effectiveness of existing antituberculosis drugs in the future [59].

The above factors of *Mtb* load, duration of symptoms, WBC, hemoglobin, and beta-1 globulin showed statistical significance in the single-factor analysis but were not statistically significant in multifactor analysis, potentially due to their limited contributions or confounding factors.

The present study has several limitations. First, because it was an observational study, Berkson’s rate bias could not be excluded. For example, patients with mild conditions who were not hospitalized and patients who did not seek treatment were not included, resulting in a low number of cases with an image grading of “Minimal/Mild”. Second, for several variables, more than 10% of the data were missing. However, the missing data were less likely to affect the conclusions of our study due to the potential existence of nondifferential misclassification. Third, the patients included in our analysis were all confirmed ATB patients. Therefore, the results might not be suitable for clinical diagnosis or applicable to suspected TB cases. However, in ATB patients who are HIV-negative, we defined the specific level of the positive correlation between CD4 and CD8 T-cells and for the first time provided a report on the negative association between CD4 and CD8 T-cell counts and EDCT results.

## Conclusion

In summary, examining T-lymphocyte subsets in ATB patients, especially CD4 and CD8 T-cells, is of great value in evaluating the immune function of the patients and assessing their disease status. The changes in the prevalence of the two subtypes of T-cells were negatively correlated with EDCT results, and the observed increase in the CD4/CD8 ratio suggests that other possible signs of drug resistance should be examined. For patients with hypoproteinemia or high alpha-1 globulin levels or patients of advanced age, immunological interventions in addition to anti-TB therapy can be beneficial and may help promote the development of host-directed therapies. However, these hypotheses require confirmation through in-depth, high-quality, randomized controlled trial studies.

## Electronic supplementary material

Below is the link to the electronic supplementary material.

Supplementary file1 (DOC 38 kb)

Supplementary file2 (DOC 32 kb)

Supplementary file3 (DOC 76 kb)

Supplementary file4 (DOC 32 kb)
