# Supplementary material for: Changes in T-lymphocyte subsets and risk factors in human immunodeficiency virus-negative patients with active tuberculosis
Source: Infection. 2020 May 29;48(4):585–95. doi: 10.1007/s15010-020-01451-2 (PMC7395032; doi:10.1007/s15010-020-01451-2)
Supplement: Supplementary file 1 — Supplementary file1 (DOC 38 kb) [file 15010_2020_1451_MOESM1_ESM.doc]

**Supplementary materials**

| **Supplementary Table 1** Smear grading report standard^a^ |
| --- |
| **Sputum smear** |
| +: 1–9 bacteria/50 fields; |
| 1+: 10–49 bacteria/50 fields; |
| 2+: 1–9 bacteria/field; |
| 3+: 10–90 bacteria/field; |
| 4+: ≥ 100 bacteria/field. |
| At least 50 fields were observed for the 2+ reports and at least 20 fields were observed for 3+ and above results. |
| **Sputum culture** |
| +: the actual colony count was reported, as the bacterial colony growth was less than 1/4 of the slope surface area; |
| 1+: bacterial colony growth accounted for 1/4 of the slope surface area; |
| 2+: bacterial colony growth accounted for 1/2 of the slope surface area; |
| 3+: bacterial colony growth accounted for 3/4 of the slope surface area; |
| 4+: bacterial colony growth accounted for entire the slope surface area. |
| ^a^ Refers to *Diagnostic Criteria and Principles of Management of Infectious Pulmonary Tuberculosis*（GB15987-1995）*.* |
