# Supplementary material for: Changes in T-lymphocyte subsets and risk factors in human immunodeficiency virus-negative patients with active tuberculosis
Source: Infection. 2020 May 29;48(4):585–95. doi: 10.1007/s15010-020-01451-2 (PMC7395032; doi:10.1007/s15010-020-01451-2)
Supplement: Supplementary file 2 — Supplementary file2 (DOC 32 kb) [file 15010_2020_1451_MOESM2_ESM.doc]

| **Supplementary Table 2** Chest CT scan image classification criteria^a^ |
| --- |
| **Stage 1 (minimal/mild)**  Mild to moderately dense lesions with no cavities and involving only part of one lung or both lungs. The entire range is smaller than the volume of the lung on one side above the junction between the second rib and the sternum. |
| **Stage 2 (moderate)**  Lesions were in one lung or both lungs, but the entire range did not exceed any of the following: (1) Small or moderate diffuse lesions with a distribution that did not exceed the entire area of one lung. If lesions were in both lungs, the total area of the lesions did not exceed the area of one lung. (2) Highly dense fusion lesions that did not exceed one-third of the volume of a single lung. (3) When there were cavities, the largest diameter of the cavity was less than 4 cm. |
| **Stage 3 (advanced)**  Cases in which the lesion range exceeded the range described above for moderate lesions. |
| ^a^ Seaton A, Seaton D, Leitch AG. Crofton and Douglas’s respiratory diseases. In: Leitch AG, eds. *Pulmonary Tuberculosis: Clinical Features.* 4th edition. Oxford, UK: Blackwell Press, 1989, pp. 409-410. |
