# Supplementary material for: Changes in T-lymphocyte subsets and risk factors in human immunodeficiency virus-negative patients with active tuberculosis
Source: Infection. 2020 May 29;48(4):585–95. doi: 10.1007/s15010-020-01451-2 (PMC7395032; doi:10.1007/s15010-020-01451-2)
Supplement: Supplementary file 3 — Supplementary file3 (DOC 76 kb) [file 15010_2020_1451_MOESM3_ESM.doc]

| **Supplementary Table 3** Detailed results of optimal scale regression analysis for T-lymphocytes |  | | | | | | | |
| --- | --- | --- | --- | --- | --- | --- | --- | --- |
| Factor | Beta | Standardized  Coefficients Std.Error | df | F | Sig. | Tolerance | Importance |  |
|  |  |  |  |  |  | After Transformation | Before Transformation |  |
| CD4 T-cell Count |  |  |  |  |  |  |  |  |
| Grade^a^ | -0.095 | 0.050 | 1 | 3.552 | 0.061 | 0.874 | 0.849 | 0.047 |
| EDCT^b^ | -0.021 | 0.044 | 1 | 0.235 | 0.628 | 0.828 | 0.818 | 0.010 |
| Age, years | -0.067 | 0.054 | 1 | 1.555 | 0.214 | 0.755 | 0.753 | 0.045 |
| Body mass index, kg/m^2^ | 0.124 | 0.058 | 1 | 4.505 | **0.035** | 0.769 | 0.773 | 0.110 |
| Duration of symptoms, days | -0.071 | 0.049 | 1 | 2.099 | 0.149 | 0.854 | 0.855 | 0.037 |
| White blood cell, ×10^9^/L | 0.125 | 0.068 | 1 | 3.351 | 0.068 | 0.814 | 0.820 | 0.053 |
| Hemoglobin, g/dL | -0.089 | 0.062 | 1 | 2.027 | 0.156 | 0.568 | 0.572 | -0.064 |
| Platelet, ×10^9^/L | 0.197 | 0.070 | 1 | 7.926 | **0.005** | 0.753 | 0.754 | 0.099 |
| Erythrocyte sedimentation rate, mm/H | 0.004 | 0.068 | 1 | 0.003 | 0.958 | 0.609 | 0.608 | -0.002 |
| Prealbumin, mg/L | 0.205 | 0.088 | 1 | 5.461 | **0.020** | 0.463 | 0.467 | 0.287 |
| Albumin, g/L | 0.153 | 0.084 | 1 | 3.329 | 0.069 | 0.351 | 0.351 | 0.202 |
| Alpha-1 globulin, g/L | -0.161 | 0.055 | 1 | 8.703 | **0.003** | 0.713 | 0.707 | 0.155 |
| Beta-1 globulin, g/L | 0.030 | 0.074 | 1 | 0.161 | 0.689 | 0.699 | 0.698 | 0.023 |
| CD8 T-cell Count |  |  |  |  |  |  |  |  |
| EDCT^b^ | -0.136 | 0.091 | 1 | 2.264 | 0.134 | 0.977 | 0.859 | 0.172 |
| Age, years | -0.199 | 0.058 | 1 | 11.861 | **0.001** | 0.855 | 0.816 | 0.380 |
| Body-mass index, kg/m^2^ | 0.073 | 0.064 | 1 | 1.308 | 0.254 | 0.788 | 0.787 | 0.039 |
| Hematocrit, % | -0.098 | 0.080 | 1 | 1.511 | 0.220 | 0.651 | 0.648 | -0.041 |
| Platelet, ×10^9^/L | 0.187 | 0.073 | 1 | 6.622 | **0.011** | 0.908 | 0.891 | 0.239 |
| Prealbumin, mg/L | 0.021 | 0.084 | 1 | 0.064 | 0.801 | 0.505 | 0.499 | 0.023 |
| Albumin, g/L | 0.166 | 0.098 | 1 | 2.842 | 0.093 | 0.389 | 0.388 | 0.207 |
| Beta-1globulin, g/L | -0.032 | 0.052 | 1 | 0.368 | 0.545 | 0.778 | 0.777 | -0.018 |
| CD4/CD8 Ratio |  |  |  |  |  |  |  |  |
| Drug-resistant tuberculosis | 0.110 | 0.058 | 1 | 3.532 | 0.061 | 0.994 | 0.994 | 0.127 |
| Erythrocyte sedimentation rate, mm/H | -0.074 | 0.070 | 1 | 1.104 | 0.294 | 0.719 | 0.719 | 0.115 |
| Prealbumin, mg/l | 0.184 | 0.086 | 1 | 4.514 | **0.035** | 0.468 | 0.468 | 0.459 |
| Albumin, g/l | -0.147 | 0.091 | 1 | 2.608 | 0.108 | 0.424 | 0.424 | -0.213 |
| Alpha-1 globulin, g/l | -0.144 | 0.067 | 1 | 4.611 | **0.033** | 0.764 | 0.764 | 0.303 |
| Beta-1 globulin, g/l | 0.135 | 0.064 | 1 | 4.481 | **0.035** | 0.709 | 0.709 | 0.210 |
| Statistically significant associations are marked in bold  *EDCT* extent of the disease as assessed by chest computed tomography  ^a^ Smear grading before treatment, the criteria are shown in Supplementary Table 1  ^b^ The criteria are shown in Supplementary Table 2 |  | | | | | | | |
