# Supplementary material for: Changes in T-lymphocyte subsets and risk factors in human immunodeficiency virus-negative patients with active tuberculosis
Source: Infection. 2020 May 29;48(4):585–95. doi: 10.1007/s15010-020-01451-2 (PMC7395032; doi:10.1007/s15010-020-01451-2)
Supplement: Supplementary file 4 — Supplementary file4 (DOC 32 kb) [file 15010_2020_1451_MOESM4_ESM.doc]

| **Supplementary Table 4**  Results of comparison between the degree of lesions and different cases | | | | |
| --- | --- | --- | --- | --- |
|  | Previously treated cases | New cases | χ^2^ value | *P* value |
| Stage 1,2 (minimal/moderate) (N = 84) | 12 (14.8) | 72 (37.1) |  |  |
| Stage 3 (advanced) (N = 191) | 69 (85.2) | 122 (62.9) | 13.392 | ＜ 0.0001 |
